# A case report of biclonal immunoglobulin D lambda/lambda multiple myeloma in patient with liver echinococcosis

**DOI:** 10.11613/BM.2024.020801

**Published:** 2024-04-15

**Authors:** Andrea Prce, Željka Dunđerović, Ivanka Mikulić, Vinka Mikulić, Kristina Ljubić, Ana Ćuk, Ante Bogut, Josip Petrović, Mile Volarić, Tomislav Čolak, Fila Raguž

**Affiliations:** 1 Department of Laboratory Diagnostics, University Clinical Hospital Mostar, Mostar, Bosnia and Herzegovina; 2 Clinical Biochemistry, University of Mostar, School of Medicine, Mostar, Bosnia and Herzegovina; 3 Department of Gastroenterology, University Clinical Hospital Mostar, Mostar, Bosnia and Herzegovina; 4 Department of Hematology, University Clinical Hospital Mostar, Mostar, Bosnia and Herzegovina; 5 Department of Nephrology, University Clinical Hospital Mostar, Mostar, Bosnia and Herzegovina

**Keywords:** multiple myeloma, immunoglobulin D, monoclonal gammopathy, serum protein immunofixation

## Abstract

Less than 2% of all symptomatic multiple myeloma (MM) has immunoglobulin D (IgD) as monoclonal protein. Biclonal gammopathy is much rarer. At the time of diagnosis, disease is often in advanced stage, including renal failure, anemia, hypercalcemia and lytic bone lesions. Due to the rarity of myeloma itself, but also due to the fact that anti-IgD antisera is not used in routine practice, there are only a few reports of IgD MM described in the literature. This case report describes a patient with IgD lambda MM with anemia and renal failure. Anemia, renal failure, and > 80 percent plasma cells in bone biopsy in our patient with IgD lambda MM meets International Myeloma Working Group criteria for diagnosis of MM. The patient clinical course was similar to other patients with IgD MM. The final result of serum protein immunofixation (s-IFE) showed IgD lambda and free lambda monoclonal bands. To prevent misdiagnosis, it is necessary to use anti-IgD and anti-IgE antisera whenever the serum protein immunofixation with IgA, IgM, IgG, kappa and lambda antiserums shows a kappa or lambda monoclonal band without monoclonal band in heavy chain.

## Graphical abstract

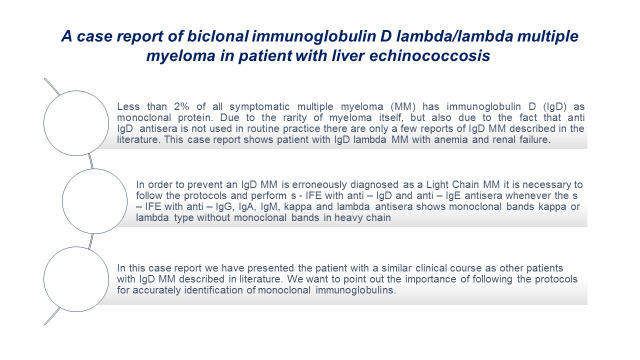

## Highlights

• Patient with IgD lambda multiple myeloma (MM) with anemia and renal failure

• It is necessary to perform serum protein immunofixation with anti-IgD and anti-IgE antisera to avoid misdiagnosis

• IgD lambda and free lambda monoclonal bands were found

## Introduction

Multiple myeloma (MM) is a disseminated malignant tumor composed of monoclonal plasma cells. It accounts for about 8% of all hematological diseases and mostly occurs in elderly and it is more common in men than women (1.4:1) (*1*).

Revised International Myeloma Working Group (IMWG) diagnostic criteria for multiple myeloma include clonal bone marrow plasma cell ≥ 10% or biopsy - proven bony or extramedullary plasmacytoma and any one or more of the following myeloma defining events that are presented in the Table 1 (*2*).

**Table 1:** Internatinal Myeloma Working Group diagnostic criteria for multiple myeloma; myeloma defining events

Evidence of end organ damage that can be attributed to the underlying plasma cell proliferative disorder	Hypercalcemia: serum calcium > 0.25 mmol/L higher than the upper limit of normal or > 2.75 mmol/L Renal insufficiency: creatinine clearance < 40 mL *per* min or serum creatinine > 177 µmol/L Anemia: hemoglobin value of > 20 g/L below the lower limit of normal, or a hemoglobin value < 100 g/L Bone lesions: one or more osteolytic lesions on skeletal radiography, CT or PET-CT
Any one or more of the following biomarkers of malignancy:	Clonal bone marrow plasma cell percentage ≥ 60% Involved: uninvolved serum free light chain ratio ≥ 100 > 1 focal lesions on MRI studies (2)
CT – computerized tomography. PET-CT – positron emision tomography-computerized tomography. MRI – magnetic resonance imaging.	

Our case report shows a monoclonal immunoglobulin D lambda type and monoclonal free light chains lambda type in a patient with anemia, renal insufficiency and clonal bone marrow plasma cell > 80%.

As a unique myeloma, immunoglobulin D (IgD) was first discovered in 1965. As a rare variant of the disease immunoglobulin D multiple myeloma (IgD MM) represents only 2% of all symptomatic myeloma. There is evidence that IgD MM is associated with high rates of kidney failure, amyloidosis, hypercalcemia and Bence Jones proteinuria (*3*-*5*).

There are two reasons why are just a few reports of IgD MM in literature, the first reason is its low concentration in plasma and the other one is the fact that antiserum for IgD is not used in routine practice. Because of its low concentration it can easily escape electrophoresis detection. Also, IgD MM is often misdiagnosed as light chain myeloma (LC MM) (*3*). In the case of IgD MM it is characterized by the high preponderance of lambda light chains over kappa light chains (*4*, *6*, *7*).

## Case report

A 69 – year- old men with liver echinococcosis was hospitalized in the gastroenterology department. Patient reached out to the doctor in primary practice because of weakened appetite and loss of 10 kilograms in a last month. The doctor ordered laboratory tests and after receiving the results of anemia and renal insufficiency, he refers him to the hospital for further diagnostic and treatment. Patient general condition was poor and he was very weak. He has liver echinococcosis for the past 30 years and was operated in 2000 and 2005. After the second operation, lung echinococcosis was also verified. Until the current hospitalization the patient had no kidney problems. As a part of diagnostic processing, samples were referred to our laboratory for routine hematology, biochemistry and urine analysis. Also, in a diagnostic process hematologist examination was requested and he ordered among other analyses serum protein electrophoresis which showed presence of M protein. After this finding he requested measurement of free light chains (FLC) kappa (κ) and lambda (λ) type both, in serum and 24 h urine, β_2_-microglobulin and serum protein immunofixation. Patient gave his consent for publishing his laboratory data.

Result of laboratory tests showed normocytic anemia accompanied with serious kidney failure. Blood cell counts were performed on Sysmex XN 1000 (Sysmex Corporation, Kobe, Japan). Immunoglobulin analyses showed that concentrations of IgA and IgM are low and IgG is towards the lower limit of the reference interval. All biochemistry tests were performed on Beckman Coulter DxC 700 AU (Danaher Corporation, California, USA).

The serum concentration of kappa and lambda light chains showed an increase in lambda light chains and a decrease in κ/λ ratio. Laboratory results are presented in Table 2. In our case altered κ/λ ratio indicate excess production of clonal FLC by the proliferating plasma cell population. A high concentration of β_2_-microglobulin as well as a high erythrocytes sedimentation rate (ESR) was in a favor of multiple myeloma. Quantitative capillary photometry for ESR was performed on iSED (Alcor Scentific, Smithfield, USA) and β2-microglobulin was measured turbidimetrically on Alinity c analyzer (Abbott Laboratories, Chicago, USA).

**Table 2:** Results of laboratory tests of a patient with IgD multiple myeloma

**Parameter**	**Result**	**Reference interval**	**Units**
**Hematology**			
WBC	11.1	3.4-9.7	x 10^9^/L
RBC	3.28	4.34-5.72	x 10^12^/L
Hb	96	138-175	g/L
Hct	0.272	0.415-0.530	L/L
MCV	82.9	83.0-97.2	fL
MCH	29.3	27.4-33.9	pg
MCHC	353.0	320-345	g/L
PLT	71	158-424	x 10^9^/L
**Biochemistry tests**			
Urea	26.8	2.8-8.3	mmol/L
Creatinine	638	64-104	μmol/L
e - GFR (CKD-EPI creatinine equation 2021)	8		mL/min/1.73m^2^
LD	285	124-241	U/L
Calcium	2.22	2.14-2.53	mmol/L
Total proteine	79.2	66-81 Hospitalized patients 60-78	g/L
Albumin	37.4	40.6-51.4	g/L
IgG	7.58	7.0-16.0	g/L
IgM	0.19	0.4-2.3	g/L
IgA	0.35	0.7-4.0	g/L
Specific tests			
β_2-_microglobulin	31.076	0.970-2.640	mg/L
FLC kappa	22.70	6.70-22.40	mg/L
FLC lambda	2320.00	8.30-27.00	mg/L
κ/λ ratio	0.01	0.31-1.56	
ESR	65	3-23	mm/3.6 ks
FLC κappa / 24 urine	13.70	1.35-24.19	mg/L
FLC lambda / 24 urine	5060.00	0.24-6.66	mg/L
κ/λ ratio / 24 urine	0.01	2.04-10.37	
M peak	21.6		g/L
**Serum protein electrophoresis**			
Albumin	36.7	40.2-47.6	g/L
	46.4	55.8-66.1	%
Alpha 1 globuline	4.8	2.1-3.5	g/L
	6.0	2.9-4.9	%
Alpha 2 globuline	6.5	5.1-8.5	g/L
	8.2	7.1-11.8	%
Beta globuline	4.4	6.0-9.4	g/L
	5.5	8.4-13.1	%
Gamma globuline	26.8	8.0-13.5	g/L
	33.9	11.1-18.8	%
A/G ratio	0.87		
WBC – white blood cells. RBC – red blood cells. Hb – hemoglobin. Hct – hematocrit. MCV – mean corpuscular volume. MCH – mean corpuscular haemoglobin. MCHC – mean corpuscular haemoglobin concentration. PLT – platelets. LD - lactate dehydrogenase. e-GFR - estimated glomerular filtration rate. ESR - erythrocyte sedimentation rate. IgG – Immunoglobulin G. IgA – Immunoglobulin A. IgM – Immunoglobulin M. FLC kappa – free light chain kappa. FLC lambda – free light chain lambda. A/G ratio – Albumine/Globuline ratio.			

Analysis of FLC in 24h urine showed Bence – Jones excretion with 7590 mg / 24h urine. Concentration of kappa and lambda light chain in both serum and urine was measured nephelometrically on BN II System (Siemens Healthcare, Marburg, Germany).

**Figure 1:**
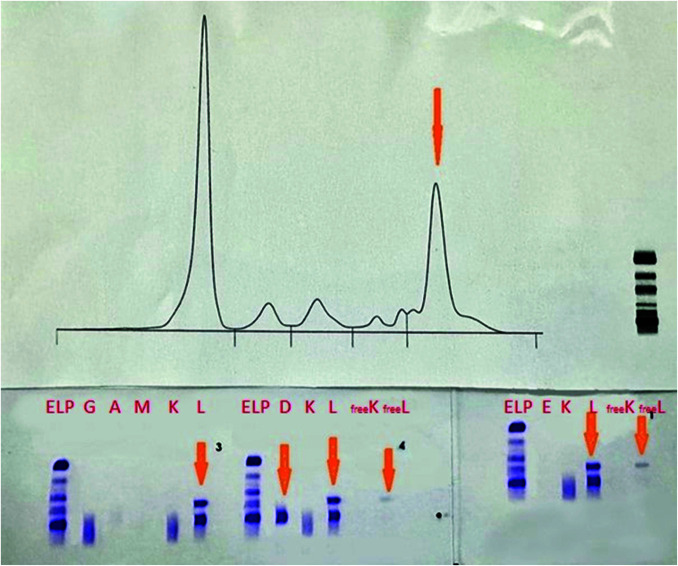
Serum protein electrophoresis and serum protein immunofixation in a patient with IgD multiple myeloma. First immunofixation (*3*): s-IFE was performed using the anti-IgG, IgA, IgM, total kappa and total lambda antisera. The arrow points two monoclonal bands lambda total light chain. Second immunofixation (*4*): s-IFE using anti-IgD, total kappa, total lambda, free kappa and free lambda antisera. The arrows points IgD monoclonal band, two monoclonal s-IFE lambda total light chain and monoclonal band free lambda light chain. Third immunofixation (*1*): s-IFE using anti-IgE, total kappa, total lambda, free kappa and free lambda antisera. The arrows points, two monoclonal s-IFE lambda total light chain and monoclonal band free lambda light chain. s-IFE - serum protein immunofixation.

Serum protein electrophoresis was performed by capillary zone electrophoresis (CZE) method using Capillarys 3 Octa (Sebia, Lisses, France). It revealed a monoclonal peak (M – spike) in the gamma globulin fraction which is showed in the Figure 1.

In order to define the class and type of monoclonal protein, after electrophoresis serum protein immunofixation (s - IFE) was performed. Immunofixation (IF) is the gold standard method to confirm the presence of a monoclonal protein. Immunofixation is a two – step procedure consisting of electrophoresis on agarose and immunoprecipitation. In the first step proteins are separated electrophoretically into sharp bands on the gel and in the second step, a specific antiserum is added to each band for a molecule of individual class and type of immunoglobulin. If there is a monoclonal synthesized immunoglobulin it will make an insoluble complex with antisera which can be stained and proved (*1*).

Serum immunofixation was performed using agarose Hydragel 2/4 IF gels on Hydrasys 2 Scan (Sebia, Lisses, France). In the first step s-IFE was performed using the anti-IgG, IgA, IgM, total kappa and total lambda antisera. The result of the first s-IFE was the presence of two monoclonal lambda total light chain without corresponding heavy chain. Following the protocol we performed s-IFE using anti-IgD, anti-IgE, total kappa, total lambda, free kappa and free lambda antisera. Serum IFE with anti-IgE antisera, total kappa, total lambda, free kappa and free lambda antisera also showed two monoclonal bands lambda total light chain without corresponding heavy chain but there was also a band on free lambda chain, which has the same electrophoretic mobility as one band in total lambda. Visualization of the agarose gel with anti-IgD antisera showed monoclonal IgD band which has the same mobility in the electric field as one of the lambda bands. The second monoclonal lambda band has the same electrophoretic mobility as free lambda chain. The final result of s-IFE showed IgD lambda and free lambda monoclonal bands (Figure 1).

M spike was measured densimetrically using orthogonally way of measurement and its concentration was 21.60 g/L (Capillarys 3 Octa, Lisses, France). After receiving results from serum protein electrophoresis and s-IFE the bone marrow examination was ordered. The bone marrow examination was performed at pathology department. Result showed hypercellular bone marrow that is extensively diffusely infiltrated with cells that are only partially recognizable as mature and mildly atypical plasma cells and mostly blastoid cells in appearance.

For further diagnostic flow cytometry immunophenotyping is recommended.

## Discussion

In this case report we presented patient with IgD lambda MM having anemia, renal failure and > 80% plasma cells in bone biopsy which meets IMWG criteria for MM diagnosis.

The rarity of IgD MM in our practice is shown by the fact that since we introduced s-IFE in our laboratory in September 2019 until today (October 2022) we had 457 requests for serum protein immunofixation and this is our first case of IgD MM. As for other types of myelomas we had: 20 IgG MM lambda type, 35 IgG MM kappa type, 4 IgM MM lambda type, 5 IgM MM kappa type, 7 IgA MM lambda type, 8 IgA kappa type, 7 LC MM lambda type and 4 LC MM kappa type.

In their study, Zagouri *et al.* states that more than 80% of IgD myeloma are lambda type, while only 38% of other myeloma have lambda light chains (*8*). The biological and pathogenetic features of the prevalence of lambda light chain in IgD MM are unclear. Also, most of the patients with IgD MM has a renal failure and significant Bence Jones proteinuria. The role of lambda light chain in kidney pathology is the subject of further research. Available data in the literature shows that patients with IgD MM have different clinical features than patients with other types of myelomas, and the exact reason for this cannot yet be determined with certainty (*8*). There are also reported a high – risk chromosomal abnormalities in patients with IgD MM but that is only on a small fraction of patients and therefore it cannot be assessed whether there are significant differences in these changes between patients with IgD MM compared to patients with another myeloma. Only a small percentage of patients with IgD MM had a detailed cytogenetics data. Current data cannot clearly say what has a greater influence on the clinical course and especially on the elevated risk of renal failure, whether it is specific cytogenetic abnormalities or tumor biology itself (*8*).

Kidney damage in multiple myeloma can be acute and chronic. Changes in the kidney tubules are dominant, while glomerulus and interstitial are much less affected. Kidney damage is caused by immunobiological activity of malignant clones, but also by deposits of immunoglobulin light chains that lead to obstruction of renal tubules and development of tubulointerstitial nephritis (*9*, *10*). Our patient, like other cases with IgD MM described in literature, had severe kidney failure. As the condition worsened, he had to undergo hemodialysis due to the high concentration of urea and creatinine.

Before development of the new drugs and autologous stem cell transplantation, survival of patients with IgD MM was shorter. With the new drugs and stem cell transplantation outcomes for these patients improved significantly (*11*, *12*). Bemelmans *et al.* in their study described the case of the patient with IgD MM with complete remission of more than eight years (*13*). Our patient general condition worsened so none of the treatments above has not started. Patient was treated with antibiotics, diuretics, electrolyte solutions and blood transfusions. Due to his previous lung problems, respiratory complications caused his death.

In order to prevent an IgD MM is erroneously diagnosed as a light chain MM it is necessary to follow the protocols and perform s-IFE with anti-IgD and anti-IgE antisera whenever the s-IFE with anti-IgG, IgA, IgM, kappa and lambda antisera shows monoclonal bands kappa or lambda type without monoclonal bands in heavy chain (*14*).

In conclusion, in this case report we have presented the patient with a similar clinical course as other patients with IgD MM described in literature. We want to point out the importance of following the protocols for accurately identification of monoclonal immunoglobulins.

## Data Availability

The data generated and analyzed in the presented study are available from the corresponding author on request.
